# A novel biomarker for predicting sepsis mortality: SCUBE-1

**DOI:** 10.1097/MD.0000000000024671

**Published:** 2021-02-12

**Authors:** Murat Erdoğan, Hüseyin Avni Findikli, İrem Okuducu Teran

**Affiliations:** a Department of Internal Medicine Intensive Care Unit, University of Health Sciences - Adana Health Practice and Research Center, Adana; b Department of Internal Medicine, Kahramanmaraş Necip Fazil City Hospital, Kahramanmaraş, Turkey.

**Keywords:** intensive care unit, mortality, SCUBE-1, sepsis

## Abstract

The mortality rate of patients diagnosed with sepsis is high. To date, many markers in sepsis patients have been studied to diagnose, determine their prognosis, and contribute to treatment. These studies were conducted in both experimental and clinical settings, but clinical trials remain limited. Therefore, more well-planned clinical studies are needed in patients with sepsis.

The current study aimed to examine the prognostic role of signal peptide-CUB-epidermal growth factor-like domain-containing protein 1 (SCUBE-1) in sepsis and sepsis-related mortality. We also wanted to study its relationship with inflammatory markers and scoring systems.

This prospective, cross-sectional, observational study included a total of 187 sepsis cases treated in the intensive care unit. Venous samples were obtained after diagnosis. The patients were separated into 2 groups: (1) the survivor group who were discharged or transferred within 28 days of the first diagnosis and (2) the nonsurvivor group who died within 28 days of the first diagnosis.

The SCUBE-1, C-reactive protein, procalcitonin, creatinine, lactate values, acute physiology and chronic health evaluation 2, sequential organ failure assessment scores were significantly higher in the survivor group, and platelets were higher in the survivor group. In addition, SCUBE-1 positively correlated with the inflammatory markers C-reactive protein, lactate, sequential organ failure assessment, and acute physiology and chronic health evaluation 2. Additionally, the SCUBE-1 value predicts 28-day mortality, and the optimal cutoff value for predicting mortality is 4,73 pg/mL.

Sepsis is a disease with high mortality. SCUBE-1 can be used as a new prognostic factor for sepsis patients.

## 1 Introduction

Sepsis is a clinical syndrome that involves physiological, biological, and biochemical abnormalities caused by a dysregulated inflammatory response to infection. Sepsis and the inflammatory response that can lead to multiple organ dysfunction syndrome and death.^[1]^ The mortality rate of patients diagnosed with sepsis is between 20% and 50%. It is one of the leading causes of death among patients in noncoronary intensive care units (ICUs).^[2]^

The severity of the disease seems to be increasing.^[3]^ It is known that the most common symptoms of severe organ dysfunction are acute respiratory distress syndrome, acute kidney failure, and extensive intravascular coagulation.^[4]^ Scoring systems are used to predict disease severity and mortality in intensive care patients, including patients with sepsis. These scoring systems are also useful for standardizing research and comparing patient care quality in ICUs. Furthermore, they are widely used worldwide. The Acute Physiology and Chronic Health Evaluation 2 (APACHE-2) scoring system is the most used scoring system in the World.^[5]^ APACHE-2. It consists of three parts: acute physiology score, age, and chronic health assessment. In this way, the acute and chronic health status of the patient is evaluated. The sequential organ failure assessment (SOFA) scoring system is used to diagnose sepsis patients in the ICU and determine their prognosis. SOFA uses simple measurements of major organ dysfunction to calculate a severity score, and it does not contain any parameters related to the patient's chronic health status.^[6]^

Signal peptide-CUB-Epidermal growth factor-like domain-containing protein 1 (SCUBE-1) is a cell surface glycoprotein that is present in platelet and endothelial cells.^[7]^ The SCUBE family has three different members. SCUBE-1 and 2 are cell-surface proteins that are expressed from platelets and endothelial cells. In particular, SCUBE-1 is highly expressed in platelets. SCUBE-1 increases platelet aggregation and adhesion. It has been identified as a potential thrombosis marker.^[8]^ SCUBE 3 is expressed during embryogenesis.^[9]^

Sepsis is a condition caused by severe inflammation that leads to microcirculation disorder, platelet activation, and endothelial damage. SCUBE-1 is a cell-surface protein that is expressed from platelets and endothelial cells so that we hypotyzed SCUBE-1 may have a role in the sepsis pathway.

Laboratory findings, especially inflammatory markers such as C-reactive protein (CRP) and procalcitonin (PCT), help in diagnosing and monitoring sepsis prognosis.

To date, many markers in sepsis patients have been studied to diagnose, determine their prognosis, and contribute to treatment. These studies were conducted in both experimental and clinical settings. However, although the majority of these studies’ preclinical results were positive, their benefits in clinical trials remained limited.

The primary purpose of this study was to investigate the prognostic role of SCUBE-1 in sepsis and sepsis-related mortality. Our other goals are to examine the relationship between SCUBE-1 with scoring systems and inflammatory markers.

### 1.1 Clinical significance

- Sepsis is a clinical syndrome caused by a dysregulated inflammatory response to infection. Sepsis and inflammatory response can lead to multiple organ dysfunction syndrome and death.
- Scoring systems and inflammatory markers are used to predict disease severity and mortality in sepsis.
- SCUBE1 is a cell surface glycoprotein that is present in platelet and endothelial cells; it also has a relationship with thrombus and endothelial dysfunction.
- The current study demonstrated that SCUBE-1 is an independent mortality predictor in sepsis patients; it also has a positive correlation with inflammatory biomarkers and APACHE II.

## 2 Material and method

### 2.1 Patients

This prospective, cross-sectional study included a total of 187 sepsis cases treated in the tertiary medical ICU of Adana City Training and Research Hospital between July 2019 and November 2019. Hospital is regional hospital and has thirty tertiary medical ICU bed. Sepsis was diagnosed according to the Sepsis-3 criteria.^[10]^ All patients aged >18 years who met the criteria were included in the study. Patients were excluded from the study if they had cancer, were pregnant, had used antibiotics in the 2 weeks before presentation at the hospital, or needed vasopressors at the time of admission. The patients were separated into 2 groups:

1. the survivor group who were discharged or transferred within 28 days of the first diagnosis and
2. the nonsurvivor group who died within 28 days of the first diagnosis.

**Figure 1:**
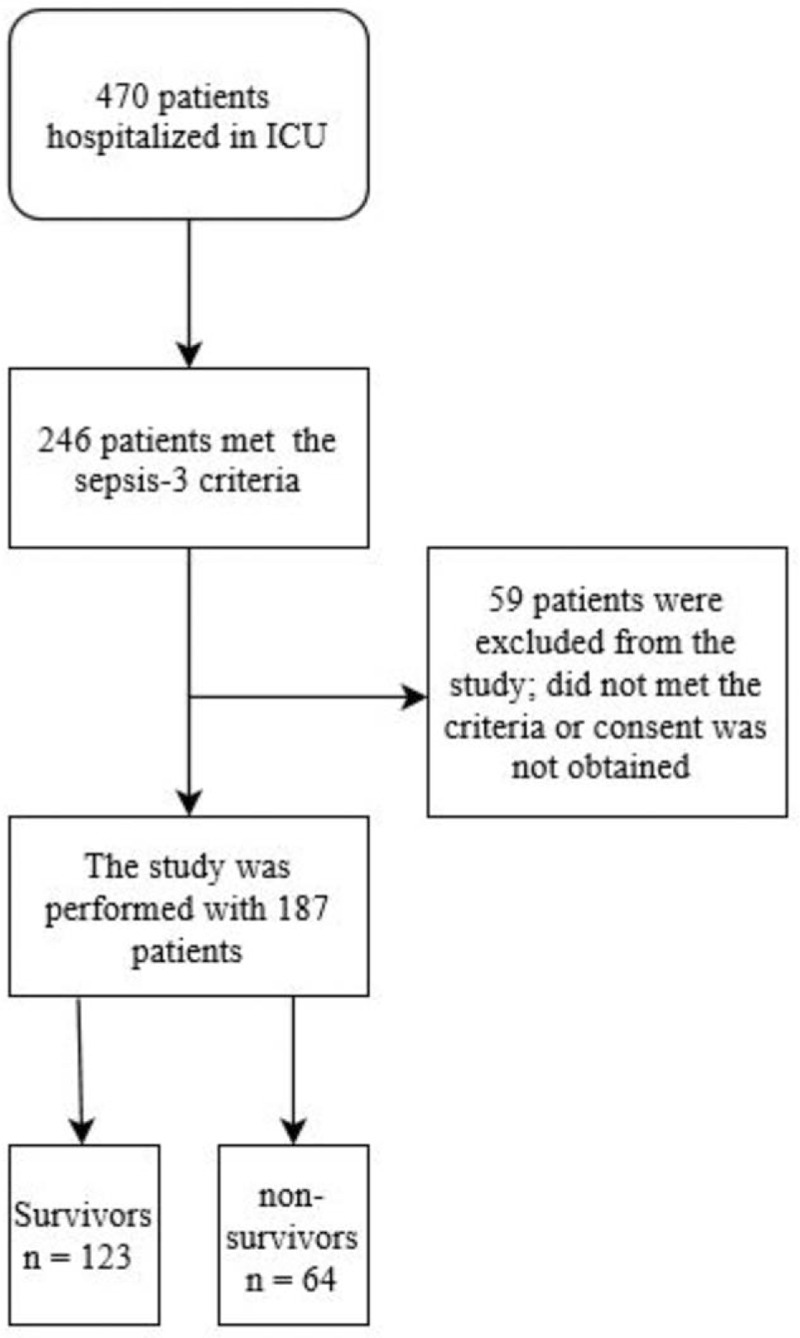
Flow diagram for SCUBE-1 studied patients.

A flow diagram for this study is presented in Figure 1. SOFA score, APACHE-2 score, and Charlson Comorbidity Index were calculated for each patient. Standard treatment was applied to all recorded patients throughout the stay in the ICU.

Approval for the study was granted by the Local Ethics Committee of Çukurova University Medicine Faculty (decision no: 88, dated: 03/05/2019). Informed consent was obtained from all patients or their legal guardians.

### 2.2 SCUBE-1 measurements

A 5 ml venous blood sample was taken from all patients after diagnosis before treatment. These venous blood samples were centrifuged at 4000 rpm for 10 min, and the serum samples obtained were frozen and stored at –80°C until assay. Biochemical analyses were performed using an autoanalyzer. SCUBE-1 levels were determined using a commercial enzyme-linked immunosorbent analysis (ELISA) kit (MyBioSource Inc, San Diego, CA).

### 2.3 Statistical analysis

Statistical analysis was performed using SPSS version 22 software (SPSS Inc, Chicago, IL). Continuous variables were expressed as mean ± standard deviation, median (interquartile range), and categorical variables as number (n) and percentage (%). The patients were separated into 2 groups: survivors and nonsurvivors. Comparisons of continuous variables between the groups were made using the Student *t* test or the Mann-Whitney *U* test according to the conformity of the data to normal distribution. The groups were compared with respect to categorical variables using the Chi-square test or Fisher exact test. Correlation coefficients of numerical variables that did not conform to at least 1 normal distribution measurement were calculated with the Pearson test and statistical significance with the Spearman test. Receiver operating characteristic (ROC) curve analysis was used to determine the sensitivity and specificity of the predictive value of SCUBE-1 for clinical outcomes. The optimal cutoff value of SCUBE-1 was derived from the Youden index. Kaplan-Meier curves were constructed using SCUBE-1 groups as strata and compared using the log-rank test. To identify risk factors for 28-day mortality, a multiple Cox proportional hazards regression model was performed.

## 3 Results

The study included 187 patients, with a median age of 74 years (interquartile range 66–80). One hundred eighty-seven of the patients, 43.2% were male. The survivors group included 123 patients and 64 nonsurvivors. The mortality rate was determined to be 35.1%. The groups had similar characteristics in terms of gender distribution (*P* = .641). When the groups were analyzed in terms of age, the nonsurvivor group consisted of older individuals (*P* = .007). According to the clinical parameters, the median APACHE-2 (*P* < .001) and SOFA score (*P* < .001) were higher in the nonsurvivor group, and no difference was determined with respect to the Charlson comorbidity index (*P* = .145). The demographics and clinical characteristics of all study patients are shown in Table 1.

**Table 1:** Baseline characteristics of patients according to mortality status.

Variables	Survivors (n = 123)	Non-Survivors (n = 64)	*P*
Sex (male, %)	52 (42.3%)	30 (46.9%)	.641
Age (yr)	71 (65–79)	77 (66–86)	.007
APACHE-2	19 (16–24)	33 (26–39)	<.001
SOFA	5 (5–8)	6 (6–12)	<.001
CCI	6 (5–9)	8 (5–10)	.145
Hb (g/dL)	10.3 (9–11.9)	10.9 (8.9–12.4)	.298
WBC (×10^3^ /μl)	11.3 (8.4–16.6)	11.5 (8.2–18.7)	.574
Plt (×10^3^/μL)	255 (161–321)	211 (159–273)	.036
Na (mmol / l)	138 (135–141)	136 (131–142)	.161
K (mmol / l)	3.9 (3.6–4.8)	4.1 (3.7–5.2)	.095
Cr	1.56 (0.97–2.65)	1.99 (1.01–4.32)	.034
Lactate (mmol/L)	16 (11–27)	20 (14–35)	.005
CRP (mg/L)	27.1 (20–53)	37.5 (21–76)	.016
PCT (g/L)	3 (0.2–21.1)	9.2 (1.9–18.7)	.036
SCUBE-1 (pg/ml)	4.12 (3.25–4.6)	5.77 (5.16–7.37)	<.001

APACHE-2 score = Acute Physiology and Chronic Health Evaluation 2 score, CCI = Charlson comorbidity index, Cr = creatinine, CRP = C-reactive protein, Hb = hemoglobin, PCT = procalcitonin, Plt = platelet count, SOFA = Sequential Organ Failure Assessment, WBC = white blood cell.

According to the laboratory parameters, the median values of creatinine (*P* = .034), lactate (*P* = .005), CRP (*P* = .016), PCT (*P* = .036), and SCUBE-1 (*P* < .001) were significantly higher in the nonsurvivor group. The mean platelet count (*P* = .036) was higher in the survivor group. The median of the other parameters was similar in both groups. The laboratory characteristics of all study patients are shown in Table 1. Spearman analysis was performed to analyze correlations between SCUBE-1 levels, sepsis severity, and biomarkers of inflammation. The results showed that SCUBE-1 levels were correlated with APACHE-II (r = 0.288, *P* < .001), SOFA (r = 0.230, *P* = .002), lactate (r = 0.159, *P* = .03), and CRP (r = 0.219, *P* = .003) (Table 2).

**Table 2:** Correlations of serum SCUBE-1 levels with clinical parameters and blood markers.

Parameter	Spearman correlation *r* value	*P* value
Age (yr)	0.104	.157
APACHE-2	0.288	<.001
SOFA	0.230	.002
WBC	0.048	,518
Plt (×10^3^/μL)	0.075	.309
Cr	0.035	.639
K	0.052	.476
Lactate (mmol/L)	0.159	.030
CRP (mg/L)	0.219	.003
PCT (g/L)	0.084	.251

APACHE-2 score = Acute Physiology and Chronic Health Evaluation 2 score, Cr = Creatinine, CRP = C-reactive protein, PCT = procalcitonin, Plt = Platelet count, SOFA = Sequential Organ Failure Assessment, WBC = White Blood Cell.

To investigate potential prognostic factors for 28-day mortality in septic patients, univariate and multivariate Cox proportional hazards regression analyses were performed using the potential risk factors (Table 1). The univariate Cox analysis indicated that the APACHE II score, levels of SOFA, platelet, lactate, CRP, and SCUBE-1 were 6 potential prognostic factors for septic patients (Table 3). All variables with a value <0.1 in univariate analyses were included in the multivariate analysis. APACHE II score (CI: 1.019–1.088; HR = 1.053; *P* = .002), SOFA (CI: 1.006–1.199; HR = 1.086; *P* = .037), CRP (CI: 1.000–1.019; HR = 1.010; *P* = .043), and SCUBE-1 (CI: 1.178–1.504; HR = 1.331; *P* < .001) were 4 independent prognostic factors for 28-day mortality in septic patients.

**Table 3:** Univariate hazard ratios of variables for predicting intensive care unit mortality and independent predictors of intensive care unit mortality by multivariate cox regression analysis.

	Univariate hazard ratios			Multivariate hazard ratios		
Variables	HR	95% CI	*P*	HR	95% CI	*P*
Age (yr)	1.012	0.990–1.035	.275			
APACHE-2	1.086	1.057–1.116	<.001	1.053	1.019–1.088	.002
SOFA	1.181	1.105–1.261	<.001	1.086	1.006–1.199	.037
Plt (×10^3^/μL)	0.997	0.995–1.000	.026			
Cr	1.117	0.981–1.273	.096			
Lactate (mmol/L)	1.025	1.006–1.043	.008			
CRP (mg/L)	1.012	1.003–1.020	.005	1.010	1.000–1.019	.043
PCT (g/L)	1.002	0.990–1.013	.783			
SCUBE-1 (pg/ml)	1.523	1.364–1.702	<.001	1.331	1.178–1.504	<.001

APACHE-2 score = Acute Physiology and Chronic Health Evaluation 2 score, Cr = Creatinine, CRP = C-Reactive Protein, PCT = Procalcitonin, Plt = Platelet count, SOFA = Sequential Organ Failure Assessment.

**Figure 2:**
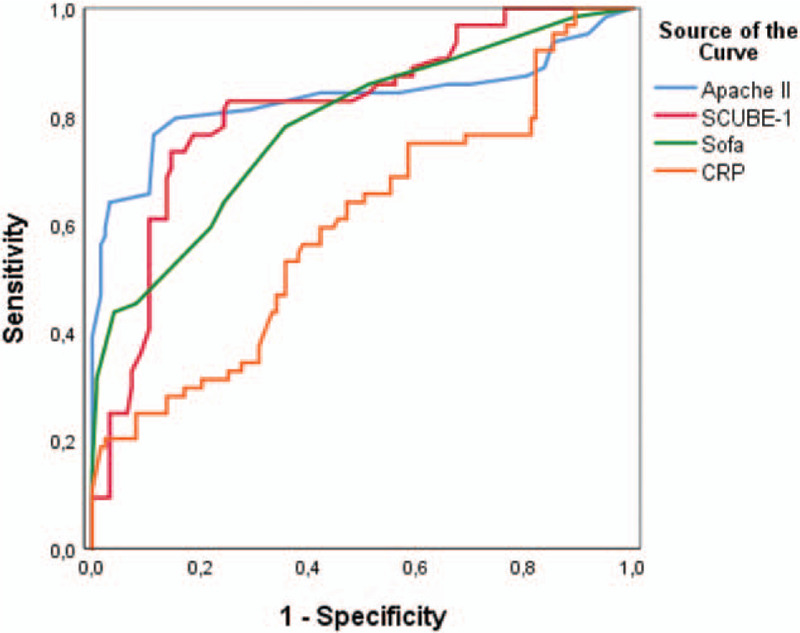
Receiver operating characteristic curve with area under the curve for predicting intensive care unit mortality.

As shown in Figure 2, baseline SCUBE-1 was a significant predictor of 28-day mortality with an area under the curve (AUC) of 0.813 (*P* < .001; CI: 0.747–0.879), a cutoff value of ≥4,73, a sensitivity of 81.3%, and a specificity of 75.6%, respectively.

Independent prognostic factors (APACHE II scores, SCUBE-1 levels, SOFA scores, and CRP levels) for risk of 28-day mortality were included in a ROC curve analysis. In terms of cut of value, AUC, *P*-value, sensitivity, and specificity. ROC curve analysis showed that APACHE II (AUC: 0.832 *P* < .001; 0.755–0.908) cut-off value of ≥24.5 a sensitivity of 79.7%, and a specificity of 84.6%, SCUBE-1 (AUC: 0.813, *P* < 0.001; CI: 0.747–0.879) a cut-off value of ≥4.73 a sensitivity of 81.3%, and a specificity of 75.6%, SOFA (AUC: 0.784, *P* < .001; 0.712–0.855) cut-off value of ≥6.50 a sensitivity of 64.1%, and a specificity of 74.6%, CRP (AUC: 0.598, *P* = .028; 0.511–0.686) cut of value of ≥29.6 a sensitivity of 59.4%, and a specificity of 57.7%, could predict increased risk of 28-day mortality in sepsis patients (Fig. 2). The AUC for all parameters was >0.5. APACHE II and SCUBE-1, respectively, had the highest predictivity.

**Figure 3:**
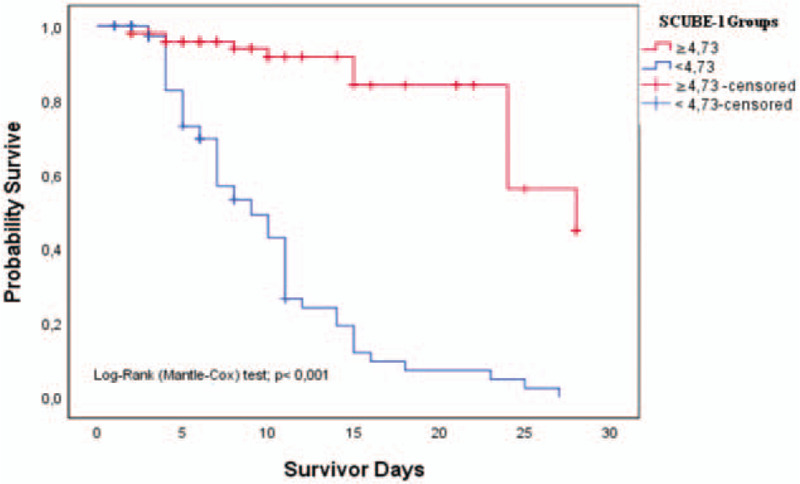
Survival curve of patients with SCUBE-1 level more than 4,73 pg/ml (red line) and those with SCUBE-1 level less than 4,73 pg/ml (blue line).

According to the cutoff value, patients were categorized into high SCUBE-1 levels (≥4,73) and low SCUBE-1 levels (<4,73). Figure 3 shows that high SCUBE-1 levels were significantly associated with a higher overall survival rate in septic patients by Kaplan-Meier curve analysis with the log-rank test (*P* < .001).

## 4 Discussion

Various biochemical markers have been explored to determine the prognosis of septic patients, but different results have been obtained. To our knowledge, this is the first study to examine the relationship between SCUBE-1 and the prognosis of septic patients. The results of this study demonstrated that SCUBE-1 is an independent prognostic factor in septic patients. In addition, SCUBE-1 showed a positive correlation with accepted inflammatory biomarkers and APACHE-2.

Many proinflammatory cytokines are released in septic patients, which causes the progression of a local infection to sepsis. Tumor necrosis factor-alpha and interleukin-1 are the primary levels, with plasma levels peaking early and eventually falling to undetectable levels. Cytokines can cause fever, hypotension, leukocytosis, and the simultaneous activation of coagulation and fibrinolysis.^[11]^ Microcirculatory injuries may result from imbalances in the coagulation and fibrinolytic systems, both of which are activated during sepsis. The increase in receptor-mediated neutrophil-endothelial cell adherence induces the secretion of reactive oxygen species, lytic enzymes, and vasoactive substances (nitric oxide, endothelin, platelet-derived growth factor, and platelet-activating factor) into the extracellular area. This is the beginning of the pathway that damages endothelial cells.^[12]^ Microcirculation injury and endothelial lesions reduce the cross-sectional area available for tissue oxygen exchange, disrupt tissue oxygenation, and cause tissue ischemia and cellular injury.

SCUBE1 is a cell surface glycoprotein that is present in platelet and endothelial cells.^[7]^ It has been previously reported that SCUBE-1 could have a role in cardiovascular, metabolic, and immunological diseases.^[8,13,14]^ Based on the increase of SCUBE-1 with platelet activation, Türkmen et al demonstrated plasma SCUBE-1 level as a potential marker for diagnosing the early stage of acute mesenteric ischemia.^[15]^ Also, Dai et al demonstrated that plasma SCUBE-1 level was significantly elevated in acute coronary syndrome and acute ischemic stroke.^[14]^ Bayoglu et al demonstrated that SCUBE1 could be a potential marker for the early detection of fetoplacental endothelial dysfunction and related maternal and fetal complications.^[8]^ Previous studies have demonstrated the relationship between SCUBE-1 and thrombus, endothelial dysfunction. Since sepsis is a condition caused by severe inflammation that leads to microcirculation disorder, platelet activation, and endothelial damage, we examined the relationship between SCUBE-1 and sepsis disease in our study.

Our study's outcomes supported our hypothesis; the SCUBE-1 level was found to be higher in non-surviving patients than in survivors. According to our study, the high plasma level of SCUBE-1 indicates that the disease will progress severely. SCUBE-1 has prognostic importance in patients with sepsis. SCUBE-1 values exceeding ≥4.73 pg/mL predict 28-day mortality.

We also studied biochemical and inflammatory markers; CRP, PCT, creatinine, and lactate levels were significantly higher in the nonsurvivor group; platelets were higher significantly in the survivor group. In addition to these results, it has been demonstrated that the level of SCUBE-1 positively correlates with the inflammatory marker CRP and impaired tissue oxygenation marker lactate.

Sepsis is associated with high mortality and substantial morbidity. Like in our study, in multicenter studies, up to 35% to 40% of patients with sepsis die from the condition.^[16,17]^ Predictive scoring systems are used to predict mortality in ICU patients. The APACHE II and SOFA scoring systems have been widely validated and used by many ICUs to classify sepsis severity and predict hospital mortality.^[5,6]^ In our study, APACHE-2 and SOFA scores were found to be statistically significant in patients who died. This is eventually compatible with the literature. In many studies in the literature, the prognostic value of biomarkers screened for sepsis has been compared with scoring systems. In our study, we compared SCUBE-1 with scoring systems and showed a positive correlation between APACHE-2 and SOFA.^[18,19,20]^

Our study's strengths are that SCUBE-1 predicts 28-day mortality and is correlated with other mortality predictors APACHE-2 and SOFA. It is also correlated with the proinflammatory marker CRP and impaired tissue oxygenation marker lactate.

This study has some limitations that should be discussed. The most important limitations of our study are the fact that it is a single-center study. We performed SCUBE-1 measurements from patients only after sepsis diagnosis. Making 1 measurement in this design is another limitation. In addition, there were not enough patients in some subgroups for the source of sepsis. For this reason, statistical analysis of the sepsis source was not performed. This is another limitation of our study. Finally, SCUBE-1 is a new biomarker, so it may not be possible to control all of the variables affecting SCUBE-1 levels.

## 5 Conclusion

In conclusion, SCUBE-1 levels have the potential to be used as a mortality predictor in septic patients. Serum SCUBE-1 levels are correlated with inflammatory markers, lactate, APACHE-2, SOFA, and disease severity. Nevertheless, there is a need for further studies to examine the specific role of SCUBE-1 in sepsis and sepsis-related mortality. In addition, the rate of change of SCUBE-1 levels after sepsis treatment should be examined.

## Author contributions

**Conceptualization:** Hüseyin Avni Findikli.

**Data curation:** Murat Erdogan, İrem Okuducu Teran.

**Formal analysis:** İrem Okuducu Teran.

**Investigation:** Murat Erdogan.

**Methodology:** Hüseyin Avni Findikli.

**Project administration:** Murat Erdogan.

**Resources:** İrem Okuducu Teran.

**Writing – original draft:** Murat Erdogan.

**Writing – review & editing:** Murat Erdogan.

## Abbreviations

Abbreviations: APACHE-2 = acute physiology and chronic health evaluation 2, AUC = area under the curve, CRP = C-reactive protein, ICU = intensive care unit, PCT = procalcitonin, ROC = receiver operating characteristic, SCUBE-1 = signal peptide-CUB-epidermal growth factor-like domain-containing protein 1, SOFA = sequential organ failure assessment.
